# Phage-Defense Systems Are Unlikely to Cause Cell Suicide

**DOI:** 10.3390/v15091795

**Published:** 2023-08-24

**Authors:** Laura Fernández-García, Thomas K. Wood

**Affiliations:** Department of Chemical Engineering, Pennsylvania State University, University Park, PA 16802-4400, USA; laugemis@gmail.com

**Keywords:** phage defense, toxin/antitoxin systems, abortive infection, altruism

## Abstract

As new phage-defense systems (PDs) are discovered, the overlap between their mechanisms and those of toxin/antitoxin systems (TAs) is becoming clear in that both use similar means to reduce cellular metabolism; for example, both systems have members that deplete energetic compounds (e.g., NAD^+^, ATP) and deplete nucleic acids, and both have members that inflict membrane damage. Moreover, both TAs and PDs are similar in that rather than altruistically killing the host to limit phage propagation (commonly known as abortive infection), both reduce host metabolism since phages propagate less in slow-growing cells, and slow growth facilitates the interaction of multiple phage-defense systems.

## 1. Review

In the following, the similarities between PD and TA systems are outlined in regard to their physiological role of growth inhibition during stress. For both, an argument is made against cell suicide to thwart phage infection. Note that the term ‘abortive infection’ is becoming controversial due to its vague and evolving meaning [1]; here, we use the term, as most authors currently do, to indicate cell suicide upon phage attack [2]. Furthermore, the term ‘phage defense’ is used to indicate the host cell response to extracellular phage attack, and TAs are primarily dual-gene systems encoding a toxin that reduces cell growth and encoding an RNA or protein that prevents translation of the toxin mRNA or inhibits the activity of the toxin.

### 1.1. Toxin/Antitoxin Systems Inhibit Phages without Killing the Host

TAs are categorized into eight classes based on whether they interact as proteins or RNA [3,4]. The two best-established roles for TAs are the stabilization of mobile genetic elements [5,6,7] and phage defense [8,9,10]. TAs are the most prevalent system for phage defense in that TAs are found in nearly all genomes at multiple copies, whereas restriction/modification systems are present in 90% of procaryotes [11], and CRISPR–Cas systems are present in 40% of bacteria [11]

The primary physiological role of TAs appears to be phage defense [12]. The seminal link of TAs to phage defense was through Hok/Sok, which reduced the propagation of T4 phages based on the phage halting host transcription and the activation of pore-forming toxin Hok, due the relative stability of the Hok toxin mRNA and the lack of new transcripts for the antitoxin Sok [8]. Thirteen years later, ToxN/ToxI was found to inhibit φA2 and φM1 of *Erwinia carotovora* [10]. Critically, there was no evidence of abortive infection for both Hok/Sok and ToxN/ToxI, nor have any TAs been shown to cause death at physiological doses [13]. This includes plasmid and mobile genetic element stabilization, so the term ‘post-segregational killing’ may be a misnomer as no host killing after plasmid loss has been documented [13].

Unfortunately, many authors claim that abortive infection occurs with TAs during phage defense [2,14,15,16], although there is little evidence to support this. For example, for DarT/DarG of *Escherichia coli* C7, where the toxin DarT modifies viral DNA and prevents replication as a DNA ADP-ribosyltransferase, it was argued that the mechanism of phage defense was abortive infection [17]; however, the evidence provided was weak [18] and was based primarily on the result that more phage propagation occurred as the multiplicity of infection increased, which is a trend that would hold for most phage-defense mechanisms (i.e., more phages per cell results in more lysis). Similarly, ToxN/ToxI inhibits *E. coli* from T2, T4, T5, and T6 infection due its shutoff of host transcription [19], as was found for Hok/Sok a quarter of a century previously [8]; however, the authors proposed that the mechanism was via cell death of infected cells producing ToxN/ToxI without adequate proof. In contrast to host killing, for the tripartite TAs (MqsR/MqsA/MqsC) of *E. coli* C496_10 that inhibit T2 phages [16], it was recently found that rather than killing the host, MqsR/MqsA/MqsC causes the cells to become dormant (persister) cells during T2 phage infection [20].

### 1.2. New Phage-Defense Systems Inhibit Phage without Killing the Host

As with TAs, the other less-prevalent bacterial phage-defense systems have been referred to as abortive infection/cell suicide systems [2,21,22] without adequate evidence; for example, for the Hna single-protein phage-defense system in *Sinorhizobium meliloti*, cell suicide is claimed in the title and throughout the manuscript without convincing results of abortive infection [23]. Additionally, the short Argonaute systems, which use RNA-mediated target binding to inhibit phages, were indicated to trigger cell death through NAD(P)^+^ depletion, but low copy plasmids (the physiological copy number) did not cause cell death, and death was only seen with high-copy-number plasmids (500 to 700 copies/cell), which is likely not physiologically relevant [24]. Similarly, CBASS anti-phage systems were indicated to cause cell death in the title; however, the claim of ‘death’ was based on overexpression systems in a non-native *E. coli* host [25]. In the same fashion, bacterial gasdermins were indicated to be ‘an ancient mechanism of cell death’ in the title, but ‘cell death’ was based on overproduction in a non-native host [26].

### 1.3. Fallacy of Cell Suicide during Phage Attack vs. Advantages of Dormancy

Arguments have been made about the advantages of bacterially programmed cell death during phage infection, such as circular arguments that 70% of prokaryotes encode abortive infection systems such as CBASS, Pyscar, and Theoris, as well as the CapRel and ToxN/ToxI TAs [21]; however, the systems that are counted as ‘proof’ of abortive infection based on its prevalence have not been shown to involve cell death at physiological levels of expression. Instead, rather than invoking cell death, these systems have effectors that reduce metabolism by degrading mRNA, halting translation, reducing energetic compounds such as NAD^+^ and ATP, and damaging the membrane. Similarly, it has been argued that cell suicide ‘makes biological sense’ as a means of last resort [2]; however, this view fails to incorporate the ideas of persistence and resuscitation from persistence, which is illogical since all tested cells form persister cells and persistence appears to be a universal stress response [27]; i.e., since there could be no more prevalent stress than phage attack, it is likely that a subpopulation of cells form persister cells during phage attack [20]. Furthermore, cell suicide during phage attack is inconsistent from the perspective of individual cells and has yet to be proven beneficial for kin.

Instead, it is far more likely that rather than causing cell death, which, in general, has not been shown, cells reduce metabolism during phage attack, and a subpopulation of cells enters the dormant (persister) state. The benefits of dormancy include (i) providing increased time for host-encoded phage-defense systems to function; (ii) slowing viral replication by depriving the phage of its requisite nucleotides and proteins; (iii) providing time for spacer accumulation for CRISPR–Cas [28]; (iv) increasing genetic diversity [29]; and (v) reducing bacteria–phage coevolution [29].

Evidence of the first point for the advantages of dormancy includes the fact that CRISPR–Cas has been shown to act first in phage defense, followed by restriction/modification systems in *Listeria* spp. [30], and the MqsR/MqsA/MqsC TAs has been shown to work with the McrBC restriction/modification system during T2 phage attack [20]. For the second point, it is well-established that slow-growing cells produce fewer phages [31]. For the third point, CRISPR–Cas type III and VI systems induce dormancy by degrading host RNA, and the very existence of spacers in all CRISPR–Cas systems demonstrates that cells must survive phage attack. Similarly, growth-inhibited cells have more spacers [32]. The fourth and fifth points have been established for bacteria that form spores during phage attack [29], and since persister cells are dormant [33], it should hold for them as well.

## 2. Perspectives

**Figure 1:**
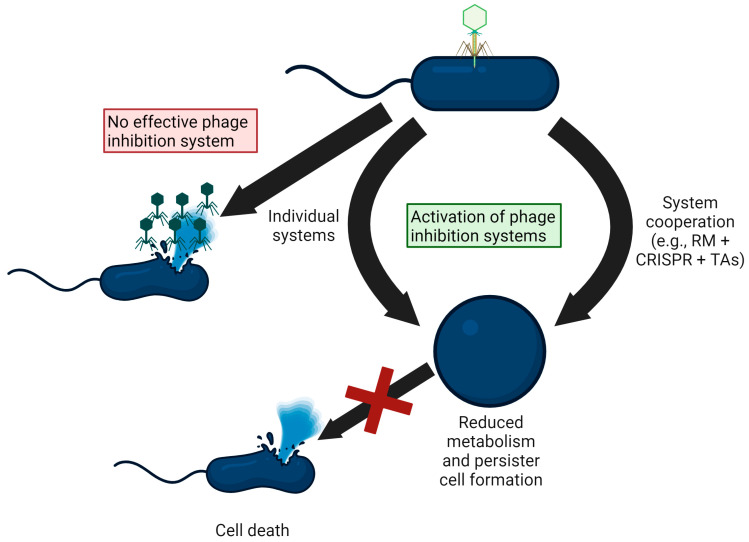
Schematic showing phage defense systems reduce metabolism to successfully thwart phages, rather than causing cell death. At high multiplicity of infection (MOI, left), most bacteria succumb to phage attack, although usually there are cells that survive that have mutations or are persisters that are metabolically inactive. For cells with active phage-defense systems, reduced metabolism is more likely than cell death, and some of the cells with reduced metabolism survive phage attack and resume metabolism (including persister resuscitation) once the phages are cleared and nutrients are provided. ‘RM’ refers to restriction/modification; ‘TAs’ refers to toxin/antitoxin systems.

If both phage-defense systems and TAs are primarily used for growth reduction rather than for cell death (Figure 1); then, one can imagine that over the billions of years of the bacteria fighting phages, bacteria have developed proteins that are toxic to every core metabolic pathway, and that these growth-inhibiting proteins have been incorporated into phage defenses to thwart phage attack; evidence of this includes the myriad toxic proteins of PDs and TAs. This also implies that there are many additional types of phage-defense systems remaining to be discovered. As their mechanisms are investigated, as outlined herein, cell suicide should not be assumed, and better experiments should be designed to investigate whether PDs induce cell death; i.e., showing that PDs are less effective at high MOI and showing less phages produced is not sufficient to conclude that cell suicide occurs. Although cell suicide during phage attack may exist, evidence-based science using current results precludes claiming the existence of cell suicide for phage defense.
